# Soil Microbial Co‐Occurrence Networks Across Climate and Land Use Gradient in Southern Italy

**DOI:** 10.1111/1758-2229.70093

**Published:** 2025-04-10

**Authors:** Mohamed Idbella, Giuseppina Iacomino, Ahmed M. Abd‐ElGawad, Giuliano Bonanomi

**Affiliations:** ^1^ AgroBioSciences (AgBS) Program, College of Agriculture and Environmental Sciences Mohammed VI Polytechnic University Ben Guerir Morocco; ^2^ Department of Agricultural Sciences University of Naples Federico II Portici Italy; ^3^ Plant Production Department College of Food & Agriculture Sciences, King Saud University Riyadh Saudi Arabia; ^4^ Task Force on Microbiome Studies University of Naples Federico II Naples Italy

**Keywords:** agroecosystems, microbiota, natural ecosystems, network modularity, next‐generation sequencing, soil chemistry

## Abstract

Despite extensive research on microbiota across land use gradients, it remains unclear if microbial co‐occurrence relationships exhibit consistent patterns. Here, we assessed microbial co‐occurrence networks of seven natural ecosystems— *Quercus ilex* forest, *Fagus sylvatica* forest, *Abies alba* forest, Mediterranean and mountain grasslands, and subalpine and Mediterranean shrublands—and five agroecosystems, including vineyards, horticulture, greenhouse, a polluted agricultural system, and an arid greenhouse. Soil chemistry, such as pH, organic carbon and total nitrogen, was characterised, and soil microbiota were profiled using high‐throughput sequencing from 242 soil samples. Our results revealed that mountain grasslands had the highest organic carbon (86.4 g/kg), while the arid greenhouse had the lowest (6.1 g/kg). Mediterranean grasslands had the lowest pH of 5.79, and vineyards had the highest electrical conductivity of 0.901 dS/m. Notably, natural ecosystem networks exhibited greater modularity, with protected horticulture showing exceptionally the highest (0.937), while intensive agriculture within agroecosystems had a significantly lower modularity of 0.282. Modularity and the number of modules were positively correlated with soil P_2_O_5_, while network diameter, path length and clustering coefficient were correlated with soil pH. Additionally, edges and nodes number, average degree and microbial diversity were positively associated with organic carbon and total nitrogen. These findings highlight that natural ecosystems foster more complex and resilient microbial networks, underscoring sustainable land management's importance to preserve soil health and microbial diversity.

## Graphical abstract

Microbial co‐occurrence networks differ across ecosystems, with natural environments exhibiting greater complexity and resilience than agroecosystems. Soil properties like pH, organic carbon and nitrogen strongly influence network structure, highlighting the importance of sustainable land management for preserving soil health and microbial diversity.

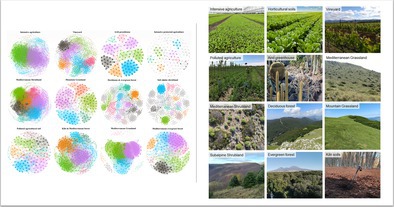

## 1 Introduction

Intensification of land use is a significant driver of biodiversity loss in the 21st century (Sala et al. 2000; Zabel et al. 2019), leaving enduring effects on ecosystems (Canelas and Pereira 2022). Transforming forests into cultivated lands often diminishes the input of organic residues, leading to a decrease in soil fertility (Galindo et al. 2022), higher soil erosion rates (Hu et al. 2021), and the depletion of soil organic matter and nutrients (Ansari et al. 2022). Intensive land use also compromises ecosystem multi‐functionality (Sünnemann et al. 2023) and exacerbates environmental issues, such as increased greenhouse gas emissions (Hong et al. 2021) and nitrogen leaching (Gao et al. 2021). Grasping the complete scope and persistence of intensive land use effects on ecosystems remains challenging. Despite clear evidence of their lasting nature, the detailed mechanisms and durations are not entirely known.

Changes in land use, such as deforestation for agriculture and livestock, are associated with intensification of tillage regime, fertilisation and pesticide use, which may profoundly affect the soil microbiome over time (Bonanomi et al. 2011). These changes influence plant communities and management practices, altering the availability, timing and spatial distribution of substrates for microbial life (Cai et al. 2018). The effects of land use changes and management practices on soil microbial communities have been extensively studied in forest and grassland ecosystems, often comparing tilled and fertilised systems (Dong et al. 2023; Cornell et al. 2023; Idbella and Bonanomi 2023; Zhang et al. 2024). For instance, historical tillage practices, implemented 60 years prior to conversion to longleaf pine savanna, have been shown to impact the diversity and composition of bacterial and fungal communities differently (Turley et al. 2020). Additionally, perennial and annual cropping systems offer distinct rhizosphere inputs, influencing fungal communities and total microbial biomass (Liang et al. 2012; Zhang et al. 2024). Grazing and fertilisation also modify soil pH, salinity and resources, thereby affecting the abundance of microbial groups involved in nitrogen cycling (Meyer et al. 2013). While the impacts of land use changes on the composition and diversity of soil microbial communities are widely studied, the understanding of how these drivers affect the number and intensity of interactions among community members remains limited. This is partly due to the inherent challenge of directly observing or measuring microbes and their interactions.

Soil microbial communities form complex networks of ecological relationships, encompassing parasitism, mutualism, neutralism, predation and competition (Faust and Raes 2012). These relationships play a pivotal role in determining the stability and resilience of microbial communities in the face of disturbances (Bardgett and Caruso 2020; Wu et al. 2021). Co‐occurrence networks have emerged as valuable tools for representing these complex relationships (Oña et al. 2025), enabling the exploration of species associations and the assessment of their complexity and stability based on topological properties (Yuan et al. 2021). Studies by Morriën et al. (2017) and Lupatini et al. (2014) revealed that cropped soils, disturbed by agricultural practices, exhibited the least complex bacterial networks compared to pastured or forest soils. Additionally, DeVries et al. (2018) observed that drought‐induced changes in vegetation composition and soil water availability led to destabilisation properties in soil bacterial networks. Shi et al. (2016) found that networks of rhizosphere microbes in a greenhouse microcosm experiment were more complex than those in surrounding soils, highlighting the influence of plant‐microbe interactions on network complexity. Despite numerous studies examining the effects of agricultural practices on soil microbial co‐occurrence networks, deciphering these networks remains challenging due to the difficulty in distinguishing true biological relationships from noise and identifying the key drivers of community dynamics.

In this study, we investigated the impact of land use intensity on soil microbial co‐occurrence ecological networks by comparing seven natural ecosystems—including *Quercus ilex* evergreen forest, *Fagus sylvatica* deciduous forest, *Abies alba* evergreen montane forest, Mediterranean and mountain grasslands, subalpine and Mediterranean shrublands—with five agroecosystems, namely vineyard, horticultural cultivation in open fields and greenhouses, a polluted agricultural system, and a *cactus* greenhouse arid ecosystem. Our hypothesis posits that variations in soil chemical properties and the intensity of agrochemical use, particularly fertilisers and pesticides, contribute to significant differences in soil microbial networks across ecosystems. Specifically, we anticipate that agricultural ecosystems would exhibit less complex networks compared to natural ecosystems due to disruptions in microbiota stemming from land use management practices. Specific hypotheses are:

- Agricultural ecosystems would exhibit less complex, less stable soil microbial co‐occurrence networks compared to natural ecosystems due to intensive agrochemical use and altered soil chemical properties.
- Variations in soil chemical properties, such as pH, organic matter content and nutrient levels, driven by differing land use intensities, will significantly influence network complexity.

## 2 Methods

### 2.1 Study Sites Description and Soil Sample Collection

**FIGURE 1:**
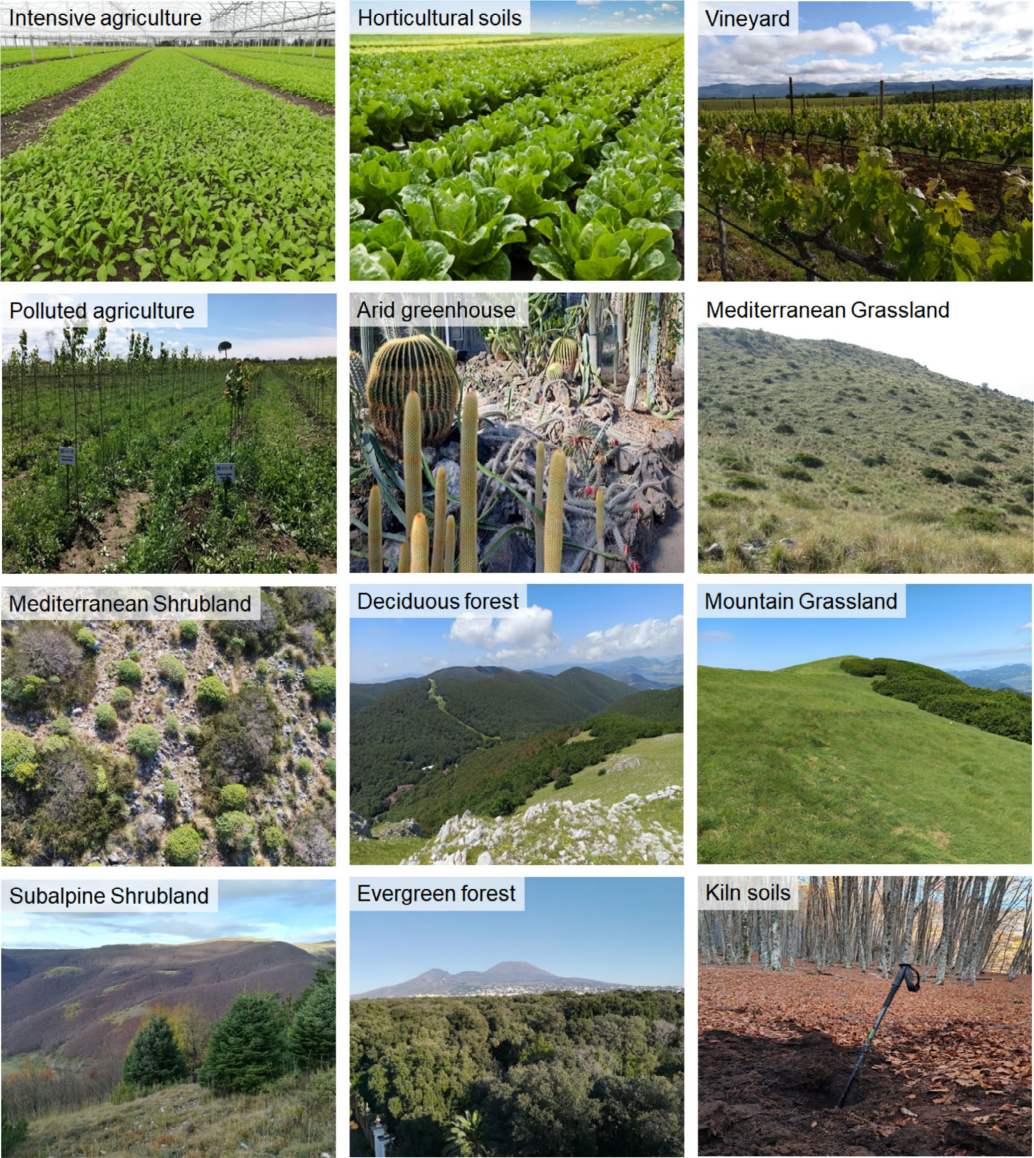
Images of selected ecosystems in the Campania Region (Southern Italy), showcasing a gradient of climate and land use intensity. The gradient includes variations in organic amendment input, synthetic fertilisers, and pesticide application. All photos by Giuliano Bonanomi and Mohamed Idbella.

The study was conducted in southern Italy, encompassing a diverse range of natural and agricultural ecosystems along a land use gradient (Figure S1). The selected sites represent different climatic conditions, soil types and management practices, providing a comprehensive framework for assessing microbial community structures in response to land use intensity. The natural ecosystems included a Mediterranean grassland dominated by *Ampelodesmos mauritanicus* , a Mediterranean shrubland dominated by six shrubs ( *Pistacia lentiscus* , *Juniperus phoenicea* , *Myrtus communis* , *Rosmarinus officinalis* , *Olea europaea* and *Euphorbia dendroides* ), and a mountain grassland dominated by *Brachypodium genuense*. Additionally, a subalpine shrubland dominated by *Pinus mugo* , a deciduous forest dominated by *Abies alba* and *F. sylvatica* , an evergreen forest dominated by *Quercus ilex* and *Q. pubescens*, and a deciduous forest dominated by *F. sylvatica* were included. On the other hand, the agricultural ecosystems covered a variety of land‐use intensities including an intensive horticultural ecosystem with frequent cultivation of tomatoes (*Solanum lycopersicon*), zucchini ( *Cucurbita pepo* ), lettuce ( *Lactuca sativa* ), cabbage ( *Brassica oleracea* ) and artichoke ( *Cynara cardunculus* ), as well as a vineyard under monoculture of *Vitis vinifera* . Additionally, an intensive greenhouse agricultural system focused on lettuce ( *L. sativa* ) and arugula ( *Eruca sativa* ), while a polluted agricultural system was primarily used for growing barley ( *Hordeum vulgare* ), rapeseed ( *B. napus* ) and buckwheat ( *Fagopyrum esculentum* ). Finally, an arid greenhouse within a botanical garden cultivated drought‐resistant cactus, specifically *Kroenleinia grusonii* and *Ferocactus glaucescens* (Figure 1).

The studied ecosystems exhibited a broad range of climatic conditions (Table 1), with mean annual temperatures in natural ecosystems ranging from 7.1°C in the subalpine shrubland to 17.6°C in the Mediterranean shrubland. In agricultural ecosystems, temperatures varied from 14.5°C in the vineyard to 25.0°C in the arid greenhouse. Precipitation levels also showed significant variation, from 789 mm in the Mediterranean shrubland to 1517 mm in the deciduous forest. In contrast, agricultural ecosystems experienced rainfall levels between 250 mm in the arid greenhouse and 1050 mm in the intensive horticultural ecosystem. Examining land use practices, agricultural ecosystems regularly received pesticide and fertiliser applications. Specifically, protected greenhouse soils and open horticultural soils were treated 8 to 16 times per year with copper, synthetic fungicides and insecticides, alongside a fumigation step with Metham sodium and the application of 180–250 kg ha^−1^ year^−1^ of NPK. Vineyard soils were treated 12–16 times per year with copper, sulfur and synthetic fungicides, plus 80 kg ha^−1^ year^−1^ of NPK. The polluted agricultural ecosystem contained higher quantities of heavy metals compared to other soils. In terms of tillage, intensive agricultural soils were tilled 4–8 times per year, horticultural soils 6–8 times per year, polluted agricultural soils 2–5 times per year, and vineyard soils once per year. Natural ecosystems, on the other hand, were not subjected to pesticide and fertiliser applications, except for the Mediterranean grassland, which was mowed twice a year.

**TABLE 1:** Land use type, geographical coordinated, elevation, climate (mean annual temperature and rainfall), organic input, pesticide and fertiliser application, tillage regime and cultivation type, and vegetation cover in the studied natural and agricultural ecosystems.

Ecosystem	Coordinates	Altitude (m)	Mean annual temperature (°C)	Mean annual rainfall (mm)	Pesticides application	Organic inputs and fertilisers application	Tillage regime	Cover vegetation
Intensive agricultural system	40°26′53.59″N 14°59′20.44″E	8	19.6	—	8 to 16 times application per year of copper, synthetic fungicide and insecticides. Soil fumigation with metham sodium	Crop residues (1–2 t ha^−1^ year^−1^), pelletized manure 0.5 t ha^−1^ year^−1^ 180–250 kg ha^−1^ year^−1^ of NPK.	Soil milling 4–8 times a year.	Lettuce ( *Lactuca sativa* ), rocket ( *Eruca sativa* )
Intensive horticulture	40°26′49.45″N 14°59′50.70″E	10	15.1	1050	8 to 16 times application per year of copper, synthetic fungicide and insecticides. Soil fumigation with metham sodium	Crop residues (1–2 t ha^−1^ year^−1^), pelletized manure 0.5 t ha^−1^ year^−1^ 180–250 kg ha^−1^ year^−1^ of NPK.	Soil milling 6–8 times a year	Lettuce ( *Lactuca sativa* ), tomato (*Solanum lycopersicon*), zucchini ( *Cucurbita pepo* ), cabbage ( *Brassica oleracea* )
Vineyard	40°24′06.73″N 15°0.4′33.32″ E	290	14.5	776	12 to 16 times application per year of copper, sulfur, and synthetic fungicide	80 kg ha^−1^ year^−1^ of NPK.	Soil milling once a year	*Vitis vinifera*
Polluted agricultural soil	40°55′42.829″N 14°12′11.548″E	30	18.7	750	Pollution degree (mg/kg): 25.07 of Cu, 45.67 of Cr, 25.56 of Ni, 71.24 of Zn, 14.19 of Pb, 0.17 of Cd, and 12.54 of As	Crop residues (2–3 t ha^−1^ year^−1^)	Soil milling 2–5 times a year.	*Hordeum vulgare, Brassica napus*, and *Fagopyrum esculentum* .
Arid greenhouse	40°26′53.59″N 14°59′20.44″E	9	25	250	—	—	—	*Kroenleinia grusonii* and *Ferocactus glaucescens*
Mediterranean grassland	40°19′37.81″N 15°07′22.52″E	170	14.9	1328	—	—	Mowed twice a year	Perennial bunchgrass dominated by *Ampelodes mauritanics*
Mountain grassland	41°49′49.16″N 14°10′23.40″E	1450	10.7	932	—	—	—	Brachypodium genuense
Mediterranean shrubland	40°01′33.37″N 15°16′11.37″E	105	17.6	789	—	Natural, mixed litterfall (1 t ha^−1^ year^−1^)	—	Woody shrubs: *Euphorbia dendroides* *Juniperus phoenicea, Myrtus communis, Pistacia lentiscus, Rosmarinus officinalis*
Subalpine shrubland	42°08′45.7″N 14°06′42.9″E	2143	7.1	1300	—	Natural, mixed litterfall (2.5 t ha^−1^ year^−1^)	—	*Fagus sylvatica, and Pinus mugo*.
Deciduous forest	41°49′59.74″N 14°15′56.92″E	1421	10.5	1517	—	Natural, mixed litterfall (2 t ha^−1^ year^−1^)	—	*Abies alba* and *Fagus sylvatica*
*Quercus* evergreen forest	40°24′24.40″ N 15°05′59.16″E	285	14.3	929	—	Natural, mixed litterfall (2 t ha^−1^ year^−1^)	—	Dominated by *Quercus ilex* and *Quercus pubescens*
*Fagus* kiln forest	40°24′25.19″N 15°09′24.20″E	1210	10.6	1480	—	Natural, mixed litterfall (3 t ha^−1^ year^−1^)	—	Dominated by *Fagus sylvatica*

Soil sample collection was conducted from April 2018 to July 2022 to capture seasonal diversity within microbial communities, reflecting the influence of multiple environmental factors such as temperature, precipitation and intrinsic soil properties, particularly within natural ecosystems. To avoid bias in future microbiome analyses, a standardised protocol was used for all samples throughout the collection campaign. Specifically, within each ecosystem, replicate plots of 100 m × 50 m were identified. Within each replicate plot, 500 g of soil was collected as a composite sample from five 100 g sub‐replicates taken from three different positions within the plot. Replicates were collected from various locations within each ecosystem to ensure a representative sampling of the diversity within each ecosystem. For physical and chemical parameters assessment, soil samples were taken from a depth of 15 cm using a soil auger with a diameter of 20 cm. For microbiological analyses, the auger‐hole was extended to about 30 cm to ensure the inner walls of the core were scraped within the upper 15 cm of soil. Samples were immediately placed in sterile plastic bags and transported on ice to the laboratory for processing. After sieving through a 4 mm mesh to remove plant roots and other debris, one fraction was kept at 4°C for chemical analysis, while the other fraction was stored at −80°C until DNA extraction. Due to various logistical constraints, including the difficulty of transporting heavy soil samples in high mountain areas, the number of samples collected differed among ecosystems. Specifically, for natural ecosystems, 20 samples were collected from the deciduous forest, 12 from the mountain grassland, 21 from the Mediterranean shrubland, 9 from the Mediterranean grassland, 36 from the subalpine shrubland, 6 from the evergreen forest and 12 from kiln soils. For agroecosystems, 32 samples were collected from polluted agricultural soils, 24 from intensive agricultural soils, 30 from horticultural soils, 30 from vineyard soils and 10 from the arid greenhouse ecosystem. Thus, a total of 242 soil samples were collected for this study.

### 2.2 DNA Extraction, Sequencing and Raw Data Processing

DNA was extracted from 0.5 g of soil (fresh weight) from each replicate using the DNeasy PowerSoil Kit (QIAGEN, USA) in accordance with the manufacturer's protocol. Post‐extraction, the concentration and purity of the DNA were assessed using a Nanodrop 2000 (ThermoFisher, USA) and agarose gel electrophoresis. Bacterial 16S rRNA gene (~460 bp) V3–V4 hypervariable regions were PCR amplified using primers S‐D‐Bact‐0341‐b‐S‐17 (5′‐CCTACG GGNGGCWGCAG‐3′) and S‐D‐Bact‐0785‐a‐A‐21 (5′‐GAC TACHVGGGTATCTAATCC‐3′), and fungal ITS1‐2 subunits of the internal transcribed spacers (~300 bp) were amplified using primers BITS1fw (5′‐ACCTGCGGARGGATCA‐3′) and B58S3‐ITS2rev (5′‐GAGATCCRTTGYTRAAAGTT‐3′). Purified amplicons were sequenced on an Illumina MiSeq platform, generating 2 × 250 bp paired‐end reads.

Raw demultiplexed FASTQ sequences were processed using the DADA2 pipeline (Callahan et al. 2016), which involves filtering, trimming and clustering sequences into unique amplicon sequence variants (ASVs). Specifically, sequences were trimmed to 250 bp, primers were removed, and sequences were filtered. Error rates were estimated using approximately 4 million reads, and after removing chimeras, ASVs were accurately identified through dereplication. For the fungal pipeline, adapters and low‐quality ends were initially trimmed using Cutadapt software (Martin 2011). Taxonomic assignments were performed using the SILVA database for bacteria and the UNITE database for fungi (Quast et al. 2013; Nilsson et al. 2019). Contaminants from chloroplasts and singleton ASVs were excluded, and relative abundances were recalculated.

### 2.3 Soil Chemistry

Chemical analyses were performed on soil samples dried at room temperature until they reached a constant weight. The soil pH, electrical conductivity (EC), organic carbon (OC) content, total nitrogen (TN), cation exchange capacity (CEC), and available phosphorus (P_2_O_5_) were assessed using standard methods outlined by Sparks (1999). The total limestone content was determined through a weight method involving a reaction with a strong acid, which produces CO_2_ gas. The volume of the released CO_2_ was then measured, following the protocol of LANO: NF ISO 10693.

### 2.4 Network and Statistical Analyses

Co‐occurrence networks for bacterial and fungal communities were constructed based on individual ASVs to examine potential interactions or co‐occurrence patterns among species within various ecosystems. The analysis covered communities from 12 different ecosystems. Only ASVs with a relative abundance greater than 0.1% and present in more than two samples were included. Spearman's correlation (using the Hmisc package in R) was employed to calculate pairwise correlations between ASVs. For statistical relevance, only correlations with a Spearman's *r* > 0.6 or *r* < −0.6 and *p* < 0.05 were considered significant. The network visualisation was generated using Gephi (version 0.9.2, Bastian et al. 2009), with each edge representing a significant correlation and each node representing an ASV along with the biomass node.

The network‐level topological features analysed included the number of nodes, number of edges, average degree, average path length, modularity and average clustering coefficient. In summary, higher values of features such as average degree (indicating the average number of connections each node has with other nodes in the network), clustering coefficient (showing the tendency of nodes to cluster together) and edge density (reflecting the intensity of connections among nodes) suggest a more interconnected network. Conversely, lower values of features like average path length (the average distance between all pairs of nodes in the network) indicate closer connections within the network (Barberán et al. 2012; Ma et al. 2016).

## 3 Results

### 3.1 Soil Chemistry

Soil physical and chemical properties varied significantly among the ecosystems (*p*‐value < 0.01, Table 2). Specifically, soils from intensive agricultural systems, Mediterranean grasslands and *Quercus* evergreen forests had the highest sand content. Vineyard soils exhibited the highest clay content, whereas horticultural and subalpine shrubland soils had the highest loam content. The arid greenhouse had the highest pH at 8.74, while the Mediterranean grassland had the lowest at 5.79. Vineyards showed the highest electrical conductivity at 0.901 dS/m, compared to the lowest values found in Mediterranean grasslands (0.103 dS/m) and Mediterranean shrublands (0.131 dS/m). Limestone content peaked in *Fagus* kiln forests at 91.9 g/kg but was lowest in subalpine shrublands (2.26 g/kg) and deciduous forests (3.09 g/kg). Mountain grasslands exhibited the highest organic carbon content at 86.4 g/kg, whereas the arid greenhouse had the lowest organic carbon (6.1 g/kg) and total nitrogen content (0.63 g/kg). Conversely, the highest total nitrogen content was found in Mediterranean and mountain grasslands at 9.56 g/kg and 8.54 g/kg, respectively. Horticultural soils had the highest P_2_O_5_ content at 272.2 mg/kg, while Mediterranean grasslands had the lowest at 15.3 mg/kg. Finally, cation exchange capacity was highest in mountain grasslands (53 meq/100 g) and *Fagus* kiln forests (51 meq/100 g), and lowest in the arid greenhouse (6 meq/100 g).

**TABLE 2:** Soil physical and chemical traits of different ecosystems. Values are the average of five replicates; different letters within each row indicate significant difference (Tukey post hoc test, *p* < 0.05).

Parameter	Intensive agricultural system	Intensive horticulture	Vineyard	Polluted agricultural soil	Arid greenhouse	Mediterranean grassland	Mountain grassland	Mediterranean shrubland	Subalpine shrubland	Deciduous forest	*Quercus* evergreen forest	*Fagus* kiln forest
Sand (g/kg)	959a	516c	450c	620b	384c	840a	208d	602b	200d	192d	930a	860a
Loam (g/kg)	17d	309a	200b	230b	106c	158c	275b	237b	300a	192b	66d	117c
Clay (g/kg)	24e	175c	350a	150c	24e	2f	125c	161c	100d	238b	4f	23e
pH	7.06b	7.82b	6.33c	7.3b	8.74a	5.79d	6.88c	7.16b	6.41c	7.38b	7.19b	7.32b
EC (dS/m)	0.213d	0.263d	0.901a	0.160e	0.502b	0.103e	0.207d	0.131e	0.388c	0.137e	0.274d	0.161e
Limestione (g/kg)	6.79d	47.7b	35.2c	46.8b	36.6c	5.91d	5.45d	5.78d	2.26e	3.09e	31.8c	91.9a
Organic carbon (g/kg)	14.4f	13.3f	12.7f	20.3e	6.1 g	64.5 ab	86.4a	26.3e	28.5e	63.0c	41.6d	76.1b
Total nitrogen (g/kg)	1.68d	1.96d	4.44b	3.9bc	0.63e	9.56a	8.54a	3.07c	2.12d	4.78b	4.85b	5.92ab
C/N ratio	8.6c	6.8d	2.9e	5.2d	9.7bc	6.7d	10.1b	8.6c	13.4a	13.2a	8.6c	12.9a
P_2_O_5_ (mg/kg)	107.1c	272.2a	147.4b	150.1b	127.3bc	15.3 h	27.5 g	30.8 g	29.0 g	44.7f	76.4d	61.8e
CEC (meq/100 g)	14.4e	20.1d	25.7d	34.3c	6.0f	40.3b	53.0a	37.7bc	15.7e	42.2b	36.1bc	51.0a

### 3.2 Microbial Co‐Occurrence Networks and Topological Properties

**FIGURE 2:**
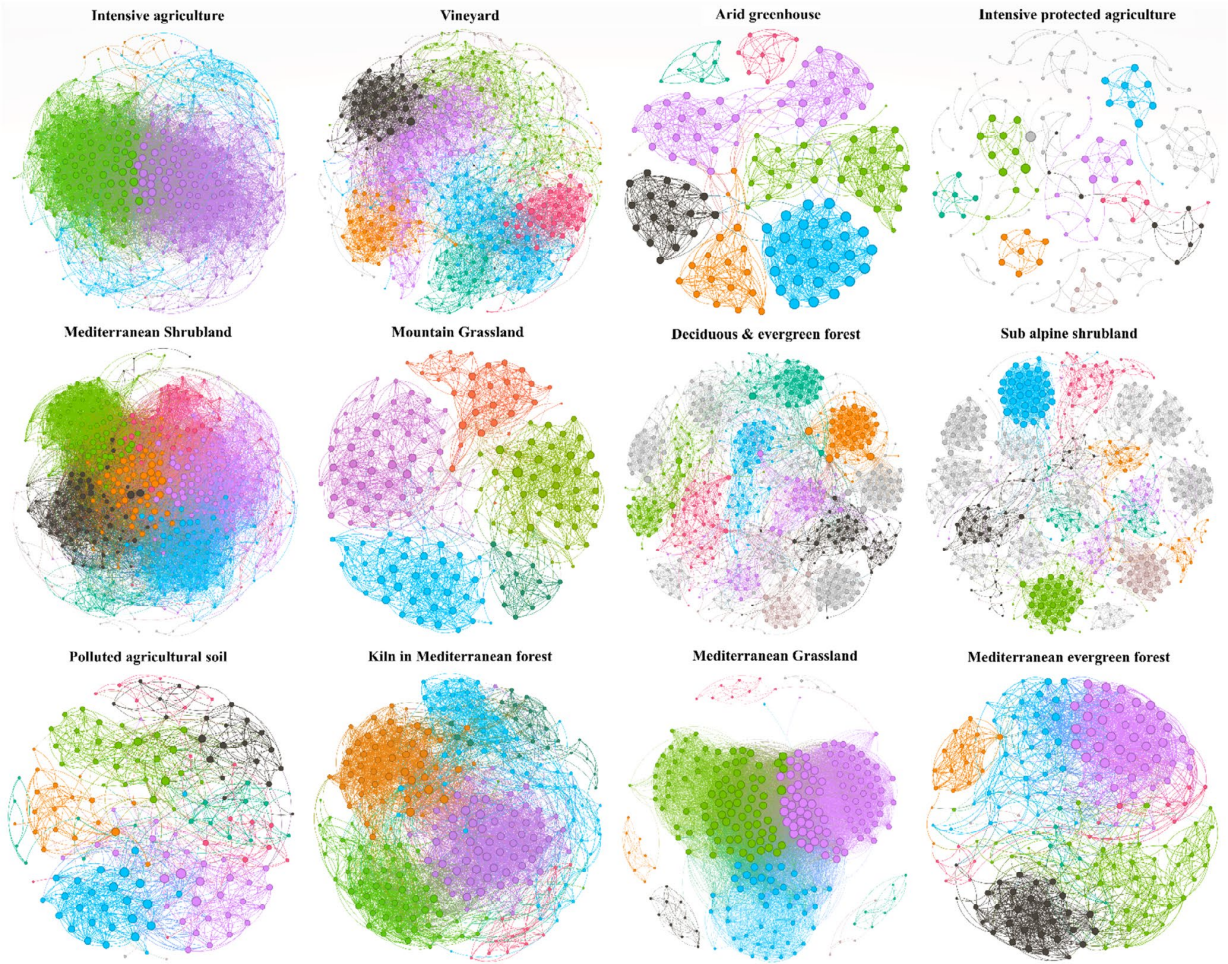
Co‐occurrence network analysis illustrating potential interactions between bacterial and fungal families across 12 ecosystems. Connections indicate strong (Spearman's ρ > 0.6 or ρ < −0.6) and significant (*p*‐value < 0.01) correlations. Node size is proportional to the weight of the connecting edges. Nodes are coloured by modularity level, representing sub‐ecosystems within each ecosystem. Module colours are independent across different ecosystems.

**FIGURE 3:**
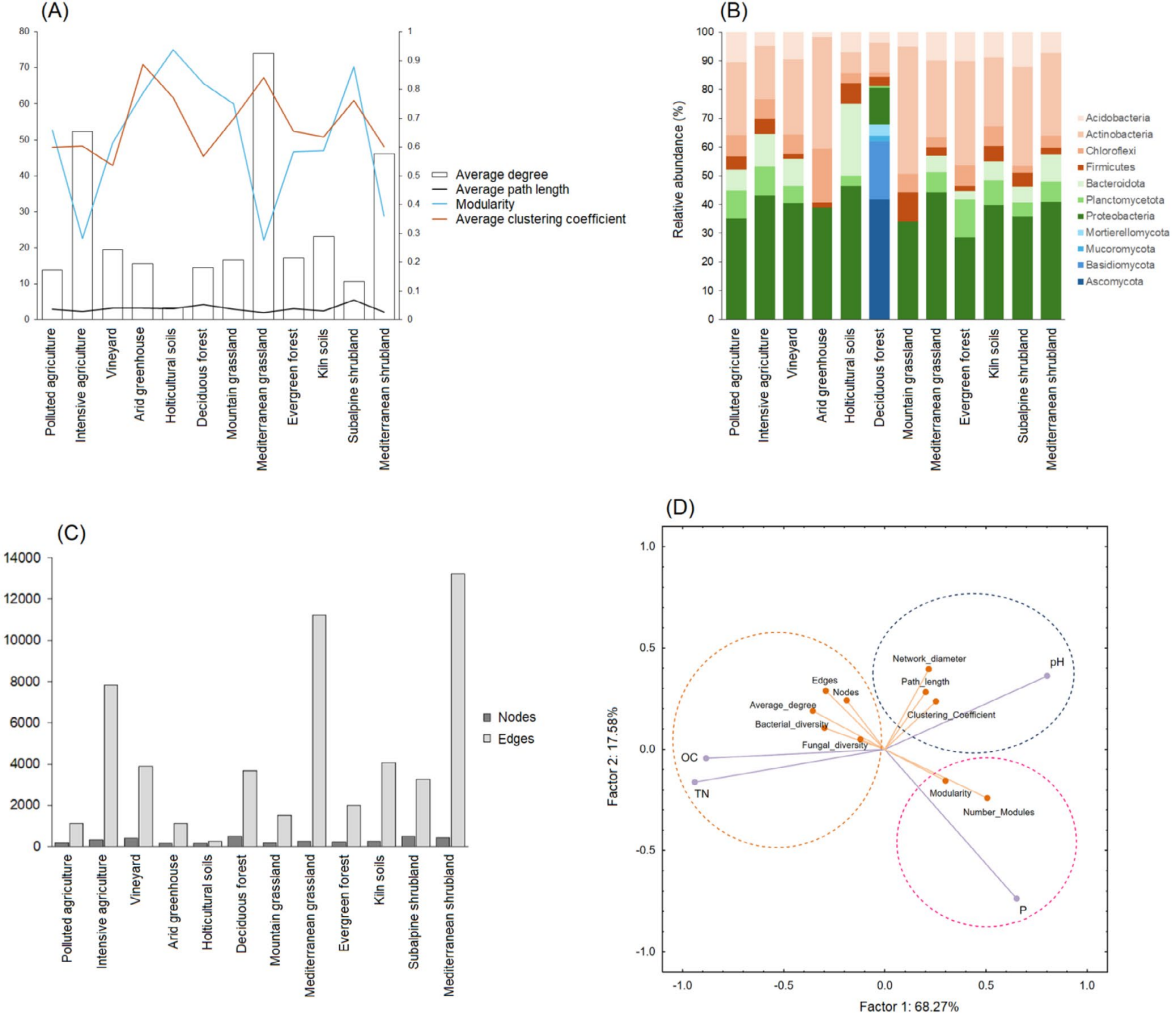
(A) Bar plot showing the topological average degree (average number of edges per node) for the networks of 12 ecosystems, with lines depicting the average path length (average number of edges in the shortest paths between all node pairs), modularity (extent to which the network can be divided into subgroups with stronger interactions), and average clustering coefficient (degree to which nodes tend to cluster together). (B) Stacked bar plot displaying the relative abundance of bacterial and fungal phyla within the networks. (C) Bar plot illustrating the number of nodes and edges in each ecosystem network. (D) Principal component analysis (PCA) showing the correlation between network topological parameters and soil pH, total nitrogen, organic carbon, and phosphorus.

To investigate the potential interactions between bacterial and fungal communities across different ecosystems with varying land use gradients, microbial co‐occurrence networks were constructed for each ecosystem based on a strong threshold matrix (−0.6 < *r* > 0.6, *p* < 0.05). Our results showed significant differences between ecosystems in terms of modularity, which measures the tendency for networks to consist of highly interconnected sub‐groups of species' nodes (Figure 2). The highest modularity index was found in the intensive protected horticultural soils at 0.937, followed by the subalpine shrubland at 0.878, the deciduous forest at 0.822, and the arid greenhouse at 0.787. The lowest modularity index was observed in the intensive agriculture at 0.282, Mediterranean grassland at 0.276, and Mediterranean shrubland at 0.359 (Figure 3A). Conversely, the average degree, representing the average number of connections each node has with other nodes in the network, was highest in the Mediterranean grassland at 73.992, followed by intensive agricultural soils at 52.253, and Mediterranean shrubland at 46.074. The lowest average degree was found in protected horticultural soils at 3.316. The average clustering coefficient, which indicates the degree to which nodes in a network tend to cluster together, was highest in arid soils at 0.887, followed by Mediterranean grassland at 0.840, and horticultural soils at 0.771. Subalpine shrubland and deciduous forest exhibited the highest average path length, indicating the average number of steps it takes to travel from one node to another across the shortest paths in the network, at 5.577 and 4.261, respectively.

The co‐occurrence networks were primarily dominated by bacterial nodes, except for the deciduous forest, which was mainly dominated by fungal nodes. Specifically, the co‐occurrence network nodes were predominantly composed of Proteobacteria and Actinobacteria. The mountain grassland network had the highest abundance of Actinobacteria, followed by the arid greenhouse and the evergreen forest. In contrast, the highest abundance of Bacteroidetes was observed in horticultural soils. The arid soil networks had the highest amount of Chloroflexi, while the deciduous forest network was dominated by Ascomycota and Basidiomycota (Figure 3B). Regarding the number of nodes and edges in the co‐occurrence networks (Figure 3C), the deciduous forest had the highest number of nodes at 507, followed by subalpine shrubland with 487 and Mediterranean shrubland with 414. The lowest number of nodes was found in the arid soil network with 144 nodes, followed by the horticultural soil network with 158 nodes. In terms of edges, the Mediterranean shrubland had the highest number with 13,241 edges, followed by Mediterranean grassland with 11,219 edges. The horticultural soil network had the fewest edges with 262, followed by the arid ecosystem network with 1,117 edges.

Results of the principal component analysis (PCA) between soil chemical properties and network topological variables explained 85.85% of the total variance (PC1: 68.27% and PC2: 17.58%). The analysis revealed specific correlations between different topological parameters and soil chemical properties (Figure 3C). Specifically, modularity and the number of modules were positively correlated with soil P_2_O_5_ content. Network diameter, path length and clustering coefficient were positively correlated with soil pH. The number of edges and nodes, average degree and bacterial and fungal diversity were positively associated with soil organic carbon and total nitrogen contents.

## 4 Discussion

Soil microbial communities exhibit diverse composition and abundance, even at a local scale (Bailey et al. 2013; Kuzyakov and Blagodatskaya 2015). These patterns are not randomly distributed but result from multiple ecological relationships occurring across different spatial and temporal scales (Lidicker 1979). In this study, we applied co‐occurrence network analysis to investigate microbial associations and their changes across different land‐use intensities. This method provides insights into microbial interactions that are otherwise not directly observable (Fath et al. 2007). Additionally, we examined how soil physical and chemical properties influence microbial networks, highlighting the role of edaphic factors in shaping microbial interactions.

The results confirm the profound influence of land use on soil properties and microbial networks. In natural ecosystems, soil properties are largely shaped by organic matter decomposition (Prescott and Vesterdal 2021), nutrient cycling (Prescott 2002) and environmental conditions (Bryanin and Sorokina 2019). Conversely, agricultural soils, due to tillage, fertilisation, pesticide application and irrigation, exhibit reduced organic carbon, increased salinity and less stable physical structure (Prager et al. 2011; Ponge et al. 2013; Tamburini et al. 2020). For example, intensive agricultural and horticultural soils contain higher sand content and less clay and loam, enhancing drainage but reducing water and nutrient retention (Wang et al. 2020; Araújo Santos et al. 2022). In contrast, natural ecosystems, such as mountain grassland and deciduous forests, retain better soil structure and higher cation exchange capacity, allowing improved water and nutrient retention (Lambooy 1984; Yunan et al. 2018; Song et al. 2022). Soil pH and nutrient availability also vary between ecosystems. Agricultural soils are typically neutral to slightly alkaline due to fertiliser use, while natural ecosystems show broader pH ranges driven by litter decomposition (Tao et al. 2019; Sellan et al. 2020). Similarly, agricultural soils have higher electrical conductivity and available phosphorus due to fertiliser use and irrigation practices (Hati et al. 2008; Ding et al. 2020), while natural soils exhibit greater organic carbon and total nitrogen from continuous organic matter input. The higher C/N ratio in natural soils indicates more stable organic matter, whereas agricultural soils, with a lower C/N ratio, experience faster decomposition and greater nitrogen availability (Fog 1988; Berg 2000; Bonanomi et al. 2021, 2023). These differences emphasise the impact of land management on soil health and underscore the need for sustainable agricultural practices that maintain microbial diversity and soil resilience.

In this study, microbial co‐occurrence networks were constructed and analysed using 242 independent samples from 12 ecosystems covering a land‐use gradient from natural to intensive agricultural environments. Our results showed that the modularity of co‐occurrence networks varies across ecosystems. Modularity, a measure of the partitioning of the network into modules or clusters, is known to be influenced by environmental variability (Liu et al. 2021). Microbial communities in natural ecosystems tend to form complex and stable networks because they have diverse microhabitats and minimal disturbances (Liu et al. 2021; Cornell et al. 2023). These networks are characterised by high modularity, indicating well‐defined subgroups of interacting microbial species (Ye et al. 2021). For example, subalpine shrublands and deciduous forests have high modularity indices, suggesting that their microbial communities are highly compartmentalised, with specific groups of microbes adapting to different environmental niches. This modularity increases the resilience of ecosystems as it allows different microbial groups to fulfil their functional roles even under changing environmental conditions (Siles et al. 2021; Gao et al. 2022). In contrast, agricultural ecosystems often have less structured microbial networks with lower modularity (Ye et al. 2021; Tian et al. 2022; Xue et al. 2022). Intensive agricultural soils, for example, have a low modularity index, indicating a more homogeneous and less compartmentalised microbial community structure. This lack of modularity may make agricultural ecosystems more vulnerable to disturbance, as the microbial communities may not have distinct groups that can buffer against environmental changes. Nevertheless, the higher modularity observed in protected horticultural ecosystems could be due to the stable and regulated environment in such ecosystems. Factors such as temperature, humidity and light are carefully controlled to optimise plant growth (Kalkhajeh et al. 2021). In addition, these ecosystems are subject to minimal external disturbance compared to open agricultural fields, which may promote the development of complex and stable microbial networks. This stability supports high modularity, as microbial communities can form and maintain specialised groups without being disturbed by external factors such as weather, pests, or human activities. On the other hand, the low modularity observed in Mediterranean grasslands and Shrublands could be due to environmental variability. Indeed, Mediterranean ecosystems are characterised by seasonal variations in temperature and moisture, which can create fluctuating conditions for microbial communities (Yuste et al. 2014; Babur et al. 2021). This variability can hinder the formation of stable and specialised microbial networks, resulting in lower modularity. In addition, Mediterranean grasslands and shrublands are subject to various disturbances, including grazing and recurrent fire, and have nutrient‐poor soils with limited organic matter, which can constrain the growth and diversity of microbial communities (Aldezabal et al. 2015; Epelde et al. 2017; Bonanomi et al. 2022).

The high average degree in Mediterranean grassland soils, indicating highly interconnected microbial networks, points to robust microbial interactions and nutrient cycling processes that are essential for maintaining soil health and productivity (Shi et al. 2016; Yuan et al. 2021; Guseva et al. 2022). Soil management practices in agricultural ecosystems significantly influence microbial interactions (Garcia‐Orenes et al. 2013; Schmidt et al. 2019; Idbella and Bonanomi 2023). Practices such as tillage, crop rotation and fertilisation can disrupt soil structure, microbial habitats and mycelial networks (Zou et al. 2015; Hartmann and Six 2022), leading to changes in microbial community composition and network properties. For example, tillage can break down soil aggregates (Álvaro‐Fuentes et al. 2008; Sarker et al. 2022), reducing the complexity of microbial habitats and altering the balance between bacteria and fungi (Young and Ritz 2000; Roger‐Estrade et al. 2010). The application of fertilisers, especially nitrogen and phosphorus, indirectly changes soil pH and may shift the composition of the microbial community towards fast‐growing, copiotrophic bacteria (Li et al. 2017; Cheng et al. 2020; Fan et al. 2021; Wang et al. 2021), which can reduce the diversity and functional stability of the microbial network. In natural ecosystems, the absence of such disturbances allows the development of more stable and diverse microbial communities (Andersen et al. 2013). The high organic carbon and total nitrogen content in mountain grasslands favour a rich microbial community, including slow‐growing, oligotrophic microorganisms that contribute to long‐term soil health and nutrient cycling (Loganathachetti et al. 2022; Villiot et al. 2023). The presence of different plant species in natural ecosystems also promotes a variety of root exudates, which serve as substrates for different microbial groups and promote microbial diversity and interactions (Idbella et al. 2022). The structure and complexity of microbial networks have a significant impact on the functioning of ecosystems (Karimi et al. 2017; Guseva et al. 2022). In natural ecosystems, the high modularity and connectivity of microbial networks facilitate efficient nutrient cycling and decomposition of organic matter. This is particularly evident in ecosystems such as deciduous forests, where fungal nodes dominate the microbial networks and play a crucial role in the degradation of complex organic compounds and nutrient recycling (López‐Mondéjar et al. 2018). The presence of Ascomycota and Basidiomycota fungi in these networks underscores the importance of fungal‐mediated processes in maintaining soil health and fertility. In agricultural ecosystems, the reduced complexity and connectivity of microbial networks can affect ecosystem functioning. Reduced modularity and lower average degree in intensive agricultural soils indicate a loss of specialised microbial functions that can affect nutrient cycling and soil fertility. The dominance of bacterial nodes, especially fast‐growing Proteobacteria, in these ecosystems indicates a shift towards more rapid, but possibly less sustainable, nutrient cycling processes (Dai et al. 2018). The reduced presence of slow‐growing, oligotrophic microorganisms in agricultural soils can lead to a decline in soil organic matter and long‐term soil health (Liu et al. 2024). The stability and resilience of ecosystems are closely linked to the structure and diversity of microbial communities (Philippot et al. 2021). Natural ecosystems with their complex and compartmentalised microbial networks are better able to withstand environmental stressors and recover from disturbances. The high modularity and connectivity of these networks enable the maintenance of important microbial functions even under changing conditions, thus supporting the resilience and sustainability of ecosystems (Mandakovic et al. 2018; Gao et al. 2022). In contrast, agricultural ecosystems, with their simplified and less interconnected microbial networks, are more vulnerable to disturbances such as climate change, soil erosion and pest outbreaks (Bonanomi et al. 2018). The frequent disturbances and intensive management practices in these ecosystems can lead to a decline in microbial diversity and functional stability, affecting soil health and productivity (Dorrough and Scroggie 2008). Sustainable agricultural practices such as reduced tillage, organic amendments and crop diversification can help restore microbial diversity and network complexity, thus improving the resilience and sustainability of agricultural ecosystems (Yan et al. 2022). Future research should focus on understanding the mechanistic links between soil management practices, microbial community structure and ecosystem functioning.

Principal component analysis of soil chemical properties and network topological variables provided important insights into the interactions within soil ecosystems. The positive correlation between modularity and the number of modules with the P_2_O_5_ content of the soil suggests that the availability of phosphorus may influence compartmentalisation within the microbial networks. This could mean that higher phosphorus levels promote the formation of specialised microbial communities that perform different ecological functions (Liu et al. 2023). Phosphorus is a critical nutrient for microbial metabolism and growth, potentially leading to more diverse and functionally distinct microbial groups that optimise phosphorus utilisation (Poeplau et al. 2016; Oliverio et al. 2020). On the other hand, the positive correlations of network diameter, path length and clustering coefficient with soil pH indicate that alkaline conditions may favour more complex and better‐connected microbial networks. The network diameter and path length provide information on the extent and efficiency of interactions within the network, while the clustering coefficient measures the tendency of nodes to form close‐meshed clusters (Barberán et al. 2012). Alkaline soils may provide a more stable and favourable environment for microbial interactions and promote extensive and efficient connectivity (Ratzke and Gore 2018). This could be due to the favourable conditions for microbial enzyme activity and nutrient availability at higher pH values, thereby promoting microbial communication and cooperation (Acosta‐Martínez and Tabatabai 2000; Šimek and Cooper 2002). The correlation between the number of edges and nodes, average degree and microbial diversity with soil organic carbon and total nitrogen content emphasises the importance of these nutrients for the formation of rich and complex microbial communities. SOC and TN are critical indicators of soil fertility and microbial biomass as they provide essential energy sources and nutrients for microbial metabolism (Adeboye et al. 2011). High levels of organic carbon and nitrogen are likely to support a diverse and dynamic microbial ecosystem with robust connectivity (edges and nodes) and interactions (average degree). This richness and complexity are crucial for maintaining soil health, nutrient cycling and ecosystem resilience. In natural ecosystems, where nutrient supply comes primarily from the decomposition of organic matter, the correlations observed in this study emphasise the role of nutrient availability in shaping microbial network structures. The presence of a diverse and interconnected microbial community is essential for efficient nutrient cycling and ecosystem stability (Van Bruggen et al. 2019). In contrast, agricultural ecosystems are often exposed to external inputs such as fertilisers, which can change the chemical properties of the soil and subsequently affect microbial networks. For example, the application of phosphorus fertiliser may promote modularity and the formation of specialised microbial communities, while the application of lime to increase soil pH could influence the connectivity and clustering of networks. The positive correlations between specific topological parameters and soil nutrients underline the central role of chemical composition in shaping microbial communities' interactions.

## 5 Conclusions

This study highlights the intricate relationships between soil microbial communities and land‐use intensity, demonstrating how soil properties and management practices shape microbial network structures. Using co‐occurrence‐based network analysis, we observed significant differences in microbial community composition, connectivity and modularity across natural and agricultural ecosystems. Natural ecosystems exhibited complex, highly modular microbial networks that enhance resilience and nutrient cycling, whereas agricultural ecosystems showed reduced modularity and connectivity, making them more vulnerable to disturbances. The observed differences underscore the impact of land management practices, such as tillage, fertilisation and irrigation, on soil microbial communities and ecosystem stability. These findings have important implications for sustainable land management. The decline in microbial network complexity in agricultural soils suggests potential long‐term risks to soil fertility and ecosystem resilience. Therefore, adopting practices that support microbial diversity, such as reduced tillage, organic amendments and crop diversification, can help restore microbial network stability and enhance soil health. Moreover, the observed correlations between soil chemical properties (e.g., phosphorus, organic carbon and pH) and microbial network parameters provide valuable insights into the role of soil nutrients in shaping microbial interactions. Future research should focus on elucidating the mechanistic links between soil management practices, microbial community dynamics and ecosystem functions. Investigating the long‐term effects of different land‐use strategies on microbial networks will be critical for developing sustainable agricultural practices. Additionally, integrating multi‐omics approaches, such as metagenomics and metabolomics, with ecological network analysis could provide deeper insights into the functional roles of microbial communities in different soil environments. Understanding how microbial networks respond to environmental stressors, such as climate change and soil degradation, will be essential for enhancing soil resilience and ensuring sustainable land use in the future.

## Author Contributions

**Mohamed Idbella:** conceptualization, investigation, writing – original draft, methodology, visualization, software, formal analysis, data curation. **Giuseppina Iacomino:** investigation, writing – review and editing, data curation. **Ahmed M. Abd‐ElGawad:** funding acquisition, writing – review and editing, validation. **Giuliano Bonanomi:** conceptualization, investigation, funding acquisition, methodology, visualization, writing – review and editing, supervision.

## Conflicts of Interest

The authors declare no conflicts of interest.

## Supporting information


**Data S1.** Supporting Information.

## Data Availability

The data that support the findings of this study are openly available in NCBI Sequence Read Archive (SRA) at https://www.ncbi.nlm.nih.gov/sra/?term=PRJNA744707, reference number PRJNA744707.
