# Supplementary material for: Soil Microbial Co‐Occurrence Networks Across Climate and Land Use Gradient in Southern Italy
Source: Environ Microbiol Rep. 2025 Apr 10;17(2):e70093. doi: 10.1111/1758-2229.70093 (PMC11985101; doi:10.1111/1758-2229.70093)
Supplement: Supplementary file 1 — Data S1. Supporting Information. [file EMI4-17-e70093-s001.docx]

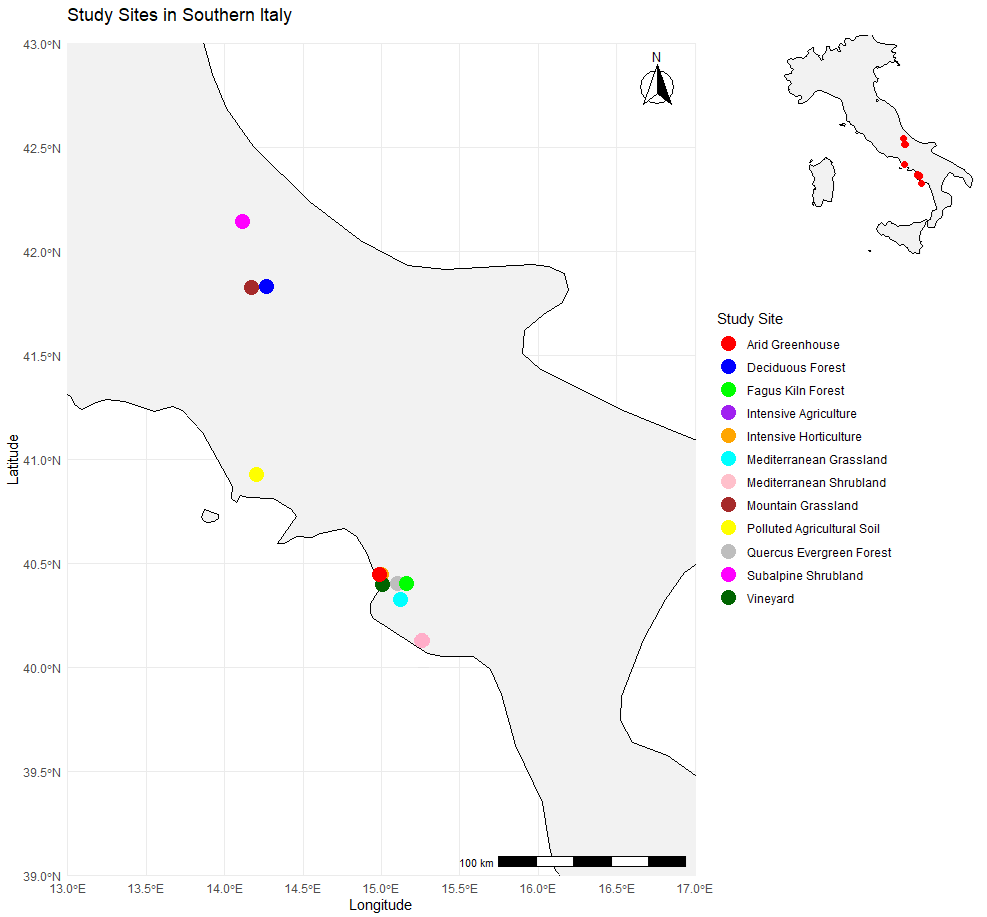


**Figure S1.** Geographical distribution of study sites in Southern Italy. The main map provides a zoomed-in view of the sampled locations, highlighting natural and agricultural ecosystems with distinct colors. The inset map in the top-right corner shows the full map of Italy with red dots indicating the study sites' locations. The scale bar and north arrow provide geographical reference.
